# Brain Metastases in Gynaecologic Cancer: A Retrospective Cohort Study Evaluating Treatment Outcomes, Prognostic Factors, and Overall Survival

**DOI:** 10.3390/curroncol31120558

**Published:** 2024-11-28

**Authors:** Carly M. Cooke, M. Ege Babadagli, Hillary Wilson, Vimoj J. Nair, Krystine Lupe, Shawn Malone, Laura Burgess, Wylam Faught, Rajiv Samant, Tien Le

**Affiliations:** 1 Division of Gynecologic Oncology, Department of Obstetrics and Gynecology, Faculty of Medicine, University of Ottawa, Ottawa, ON K1N 6N5, Canada; wfaught@toh.ca (W.F.); tle@toh.ca (T.L.); 2 Division of Radiation Oncology, Department of Radiology, Radiation Oncology and Medical Biophysics, Faculty of Medicine, University of Ottawa, Ottawa, ON K1N 6N5, Canada; ebabadagli@toh.ca (M.E.B.); hilwilson@toh.ca (H.W.); vnair@toh.ca (V.J.N.); klupe@toh.ca (K.L.); smalone@toh.ca (S.M.); laburgess@toh.ca (L.B.); rsamant@toh.ca (R.S.)

**Keywords:** brain metastases, gynaecologic malignancy, radiation

## Abstract

(1) Background: The objectives of this study were to assess survival of patients with a diagnosis of brain metastases secondary to gynaecologic malignancy and the impact of clinicopathological factors on prognosis in this population. (2) Methods: A retrospective cohort of patients with gynaecologic cancers diagnosed with brain metastases treated with radiation at a tertiary care centre from 1 January 2004 until 30 September 2023 was studied. Kaplan–Meier method and log-rank test were used to evaluate survival, and cox regression was used to identify significant predictive factors of survival. (3) Results: In total, 103 patients were included in this study. Median age at diagnosis of brain metastases was 59 (range 30–94). Median survival time following diagnosis of brain metastases was 3.6 months (range 0.4–183.8). Survival was significantly longer for patients treated with surgery combined with radiation compared to radiation alone and with stereotactic radiosurgery (SRS) compared to whole brain radiation therapy (WBRT). Cox regression revealed that primary ovarian malignancy, extracranial disease at diagnosis, and ≥3 brain metastases were associated with poorer prognosis, and complete response to prior treatment was associated with more favourable prognosis. (4) Conclusions: Data from this study will assist in providing evidence-based prognostic information to patients with gynaecologic malignancy diagnosed with brain metastases.

## 1. Introduction

Brain metastases occur in 10–30% of all cancer patients [1,2], with the most common primary site of disease being lung, breast cancer, and melanoma [3,4,5]. In contrast, brain metastases in gynaecologic malignancies are rare, with reported incidences of <2% in ovarian cancer [4,5], and <1% in endometrial and cervical cancers [5,6]. However, there is some evidence that the incidence of brain metastases associated with gynaecologic cancers is rising [5,7].

Overall, brain metastases from gynaecologic malignancies tend to be a late phenomenon in the disease course and have classically been identified as a poor prognostic factor. Following diagnosis, treatment options for metastatic brain lesions include surgical resection, radiation with whole brain radiation therapy (WBRT) or stereotactic radiosurgery (SRS), or best supportive care. There are numerous small case series assessing the efficacy of these treatment modalities and prognostic factors affecting survival following the diagnosis of brain metastases; however, these are limited in their ability to draw conclusions due to small sample size [2,8,9,10]. More recent, larger retrospective cohort studies have additionally aimed to investigate this topic; however, the data overall remain scarce [5,11,12].

The primary objective of this study was to determine the overall survival of patients with brain metastases diagnosis secondary to gynaecologic malignancy. Secondary objectives were to describe patient- and disease-related factors and their impact on survival.

## 2. Materials and Methods

**Figure 1:**
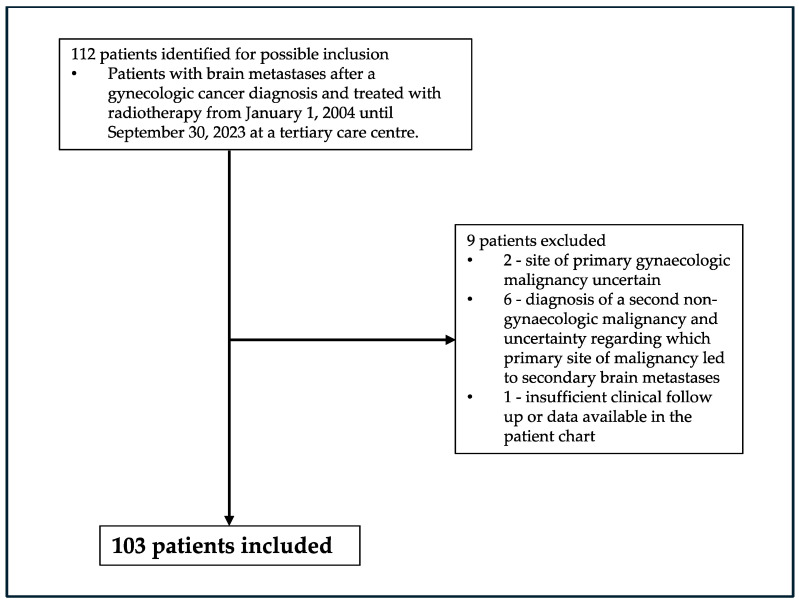
Patient inclusion flowchart.

This was a single-institution retrospective cohort study. This study was approved by The Ottawa Health Science Network Research Ethics Board (Protocol # 20230646-01H). Inclusion criteria were patients aged ≥18 with brain metastases after a gynaecologic cancer diagnosis and treated with radiotherapy at a tertiary care cancer referral centre, The Ottawa Hospital, from 1 January 2004 until 30 September 2023. Biopsy or pathologic confirmation of brain metastases was not required as long there was no known alternate primary site of malignancy and clinical and imaging data consistent with brain metastases secondary to gynaecologic malignancy. Exclusion criteria were as follows, site of primary gynaecologic malignancy uncertain, diagnosis of a second non-gynaecologic malignancy, and uncertainty regarding which primary site of malignancy led to secondary brain metastases, as well as insufficient clinical follow up or data available in the patient chart. See Figure 1 detailing the selection of patients for this study. Diagnosis of brain metastases ± was made using imaging in the form of contrast-enhanced computed tomography (CT) magnetic resonance imaging (MRI), which are supported in the literature as diagnostic modalities for brain metastases, with MRI being most sensitive [13]. Diagnosis was confirmed with pathology in patients who underwent surgical intervention.

Regarding radiation techniques used at The Ottawa Hospital for treatment of brain metastases during the study period, between 2004 and 2008, the majority of WBRT cases were treated with Cobalt-60, while for 2008 onwards, WBRT cases were primary treated with 6 MV photons using LINAC 6. Between 2004 and 2013, the majority of SRS cases were treated with 6 MV photons using LINAC, while for 2013 onwards, SRS cases were primarily treated with 6 MV photons using CyberKnife, with a handful of cases treated with 6 MV photons using either TomoTherapy or LINAC.

Factors taken into consideration when selecting patients with brain metastases for surgical intervention include solitary brain metastasis, well-controlled extracranial disease or nil, Karnofsky performance status ≥70, age < 60, mass effect/large lesion, need for diagnostic tissue biopsy, acute onset neurologic symptoms, accessible location/technically resectable, and medically operable. Surgery is typically followed by radiotherapy as postoperative radiotherapy has been shown to achieve improved control of tumours in the brain in comparison to surgery alone [14].

Data collected from patient records included age at the time of initial cancer diagnosis, primary gynaecologic malignancy, FIGO stage at the time of diagnosis, tumour grade, best clinical response to previous treatment (as defined by the Recist criteria v 1.1) [15], time from initial cancer diagnosis and from last treatment modality to diagnosis of brain metastases, imaging modality used to diagnose brain metastases, neurologic symptoms at the time of diagnosis of brain metastases, number of metastatic brain lesions, extracranial metastases (defined as metastatic disease outside the brain) on imaging at the time of diagnosis of brain metastases, site of extracranial metastases, treatment of brain metastases, and best response to treatment of brain metastases based on imaging post treatment. Descriptive statistics were used to describe the cohort’s demographics.

Median survival post diagnosis of brain metastases was measured from the date of diagnosis of brain metastases until date of death or last follow up. Median overall survival was measured from the date of initial cancer diagnoses until date of death or last follow up. The log-rank test was used to compare survival post diagnosis of brain metastases. Cox regression was used to assess survival post diagnosis of brain metastases adjusting for important clinicopathologic factors including site of primary gynaecologic malignancy, stage at initial cancer diagnosis, tumour grade at initial cancer diagnosis, brain metastases at initial cancer diagnosis, best response to prior treatment, number of brain metastases, extracranial metastases, date of treatment of brain metastases, and, additionally, based on treatment of brain metastases and the type of radiation provided. While consideration was made for inclusion of tumour histological type into our survival analyses, there was too much histologic variability amongst the 5 gynaecologic malignancies included in this study (ovary, uterus, cervix, vagina, and vulva) for meaningful analysis. The proportional hazards assumption was satisfied for the cox proportional hazards regression model used. For all statistical tests, *p* values of <0.05 indicated statistical significance. Statistics were performed using SPSS software version 29.0.2.0.

Over the study time period (2004–2023), both the number of patients referred to Radiation Oncology at our tertiary care centre with brain metastases after gynaecologic cancer diagnosis and the total number of patients referred for gynaecology malignancy were used to assess incidence of brain metastases secondary to gynaecologic malignancy in the region. Data regarding total number of patients with gynaecologic malignancy referred were unavailable for the first 5 years of this study (2004–2008). The incidence of brain metastases in patients with gynaecologic malignancy was reported over each subsequent 5-year period.

## 3. Results

A total of 103 patients met the inclusion criteria, as seen in Figure 1. Median age at the time of diagnosis of primary gynaecologic malignancy was 58 (range 29–93). Patients’ primary gynaecologic malignancy diagnosis distribution is as follows: ovary/fallopian tube/primary peritoneal (n = 46, 45%), uterine (n = 34, 33%), cervix (n = 16, 16%), and vagina/vulva (n = 7, 7%). Median age at diagnosis of brain metastases was 59 (range 30–94). Patient clinical and pathological data can be found in Table 1.

Median time from initial diagnosis to brain metastases diagnosis was 22.6 months (range 0–125). Median time from last treatment provided for their primary gynaecologic malignancy to diagnosis of brain metastases was 2 months (range 0–49). Most patients had symptoms at the time of diagnosis (n = 97, 94%), ≥3 brain metastases (n = 55, 53%), and extracranial metastases (n = 80, 78%) identified on imaging at the time of diagnosis of brain metastases. Patients were mainly treated with radiotherapy alone (n = 77, 75%); however, 26 patients (25%) additionally underwent surgery as part of their treatment for brain metastases prior to radiation therapy. Further, the most commonly used radiotherapy technique was WBRT alone (n = 64, 62%), but SRS was provided to 30 patients (29%), and 9 patients (9%) received a combination of WBRT and SRS.

Regarding the response to treatment of brain metastases, based on imaging post treatment, 13 (12.6%) patients had a complete response to treatment with no evidence of residual disease, 32 (31%) had a partial response, 3 (2.9%) had progressive disease, and 55 (53.3%) of the patients did not have imaging post treatment to quantify the response.

At the time of analysis, 91 patients had died or were lost to follow up, and 12 patients were living. Median survival time post diagnosis of brain metastases was 3.6 months (range 0.4–182.8; 95% CI 2.2, 4.9). Median overall survival from time of initial cancer diagnosis to date of death or last follow up was 34.9 months (range 0.7–228.5; 95% CI 26.1, 43.7). Table 2 shows median survival post diagnosis of brain metastases and overall survival based on patient clinical and pathological factors, with univariate analyses by the Kaplan–Meier method log-rank test.

**Figure 2:**
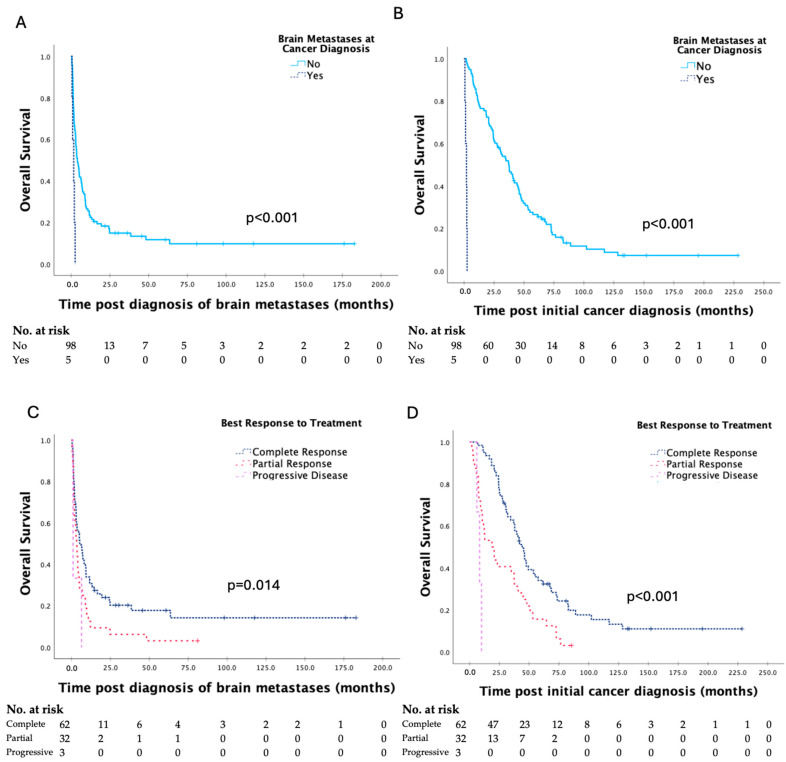

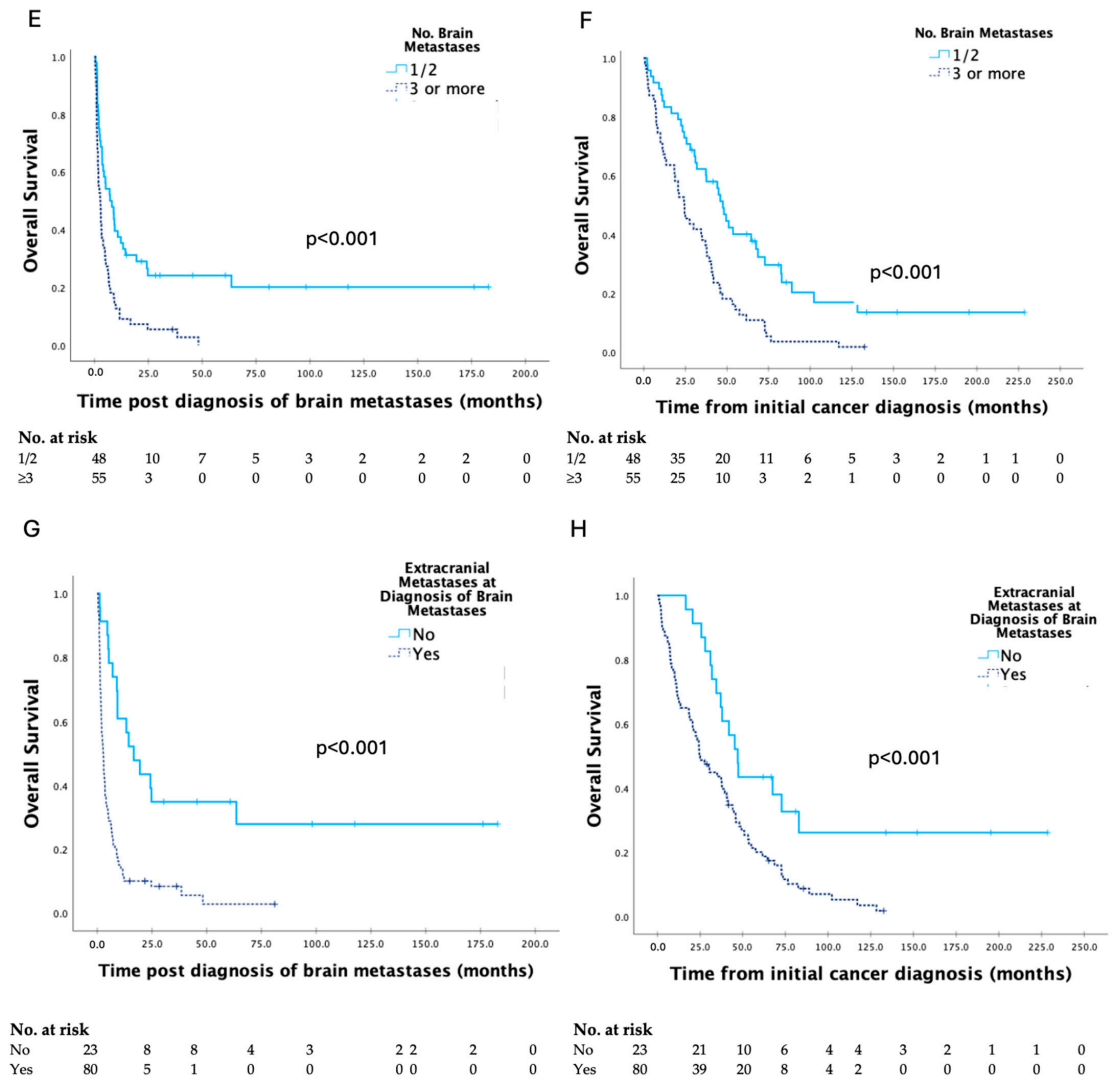
Kaplan–Meier curves for clinicopathologic factors which were shown to have a significant impact on survival post diagnosis of brain metastases and overall survival in patients with brain metastases secondary to gynaecologic malignancy, with analyses by the log-rank test (**A**–**H**).

Kaplan–Meier curves for clinicopathologic factors which were shown to significantly affect survival are shown in Figure 2. There were no significant differences in survival based on primary site of gynaecologic malignancy, FIGO stage or tumour grade at initial cancer diagnosis, or date of treatment of brain metastases. The presence of brain metastases at initial cancer diagnosis, ≥3 brain metastases, and extracranial metastases at the time of diagnosis of brain metastases were associated with significantly shorter survival, and complete response to prior treatment was associated with significantly longer survival.

**Figure 3:**
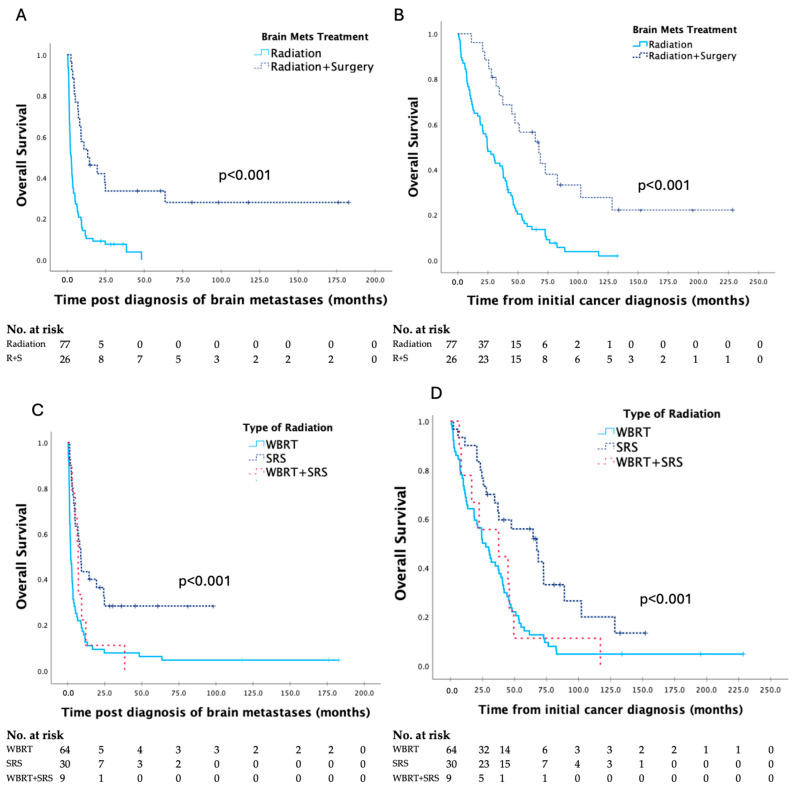
Kaplan–Meier curves for survival post diagnosis of brain metastases and overall survival in patients with brain metastases secondary to gynaecologic malignancy, based on type of treatment provided (**A**,**B**), and type of radiation provided (**C**,**D**), respectively, with analyses by the log–rank test.

Survival data based on treatment are seen in Table 3, and Kaplan–Meier curves are shown in Figure 3. Survival was significantly longer for patients treated with surgery combined with radiation compared to radiation alone and with SRS compared to WBRT.

Multivariate analysis using cox regression revealed that primary ovarian malignancy (HR 2.2, 95% CI 1.2, 3.9, *p* = 0.008), the presence of extracranial metastases at diagnosis, (HR 2.1; 95% CI 1.03, 4.3; *p* = 0.042) and ≥3 brain metastases (HR 2.0; 95% CI 1.1, 3.6; *p* = 0.029) were associated with poorer prognosis, while complete response to prior treatment (HR 0.3; 95% CI 0.1,0.9; *p* = 0.023) was associated with a more favourable prognosis following diagnosis of brain metastases. There was a trend towards improved survival in patients who were treated with surgery combined with radiation compared to radiation alone; however, this did not meet statistical significance (HR 0.5; 95% CI 0.3, 1.0; *p* = 0.07).

As seen in Table 4, based on the available data, incidence of brain metastases secondary to gynaecologic malignancy from 2009 to 2013 was 1.3%; from 2014 to 2018 was 1.6%; and from 2019 to 2023 was 1.7%, hence rising 37% (*p* = 0.26) over this time frame.

## 4. Discussion

The primary objective of this study was to assess the survival in patients with brain metastases secondary to gynaecologic malignancy, and our results showed that survival of patients with brain metastases secondary to gynaecologic malignancy remains poor, with median survival time post diagnosis of brain metastases of 3.6 months (range 0.4–182.8).

The current literature provides evidence of increasing median survival post diagnosis of brain metastases in patients with gynaecologic malignancy [2,5,8]. In one of the more recent retrospective reviews, median survival time in the ovarian cancer group was 12.5 months [5]. Cagino et al. reported median survival post diagnosis of brain metastases of 16.9 months in a small cohort of 30 patients with primary gynaecologic malignancies [8]. Further, Kim et al. showed a particularly long median survival of 28 months, likely related to the high proportion of patients with more favourable disease characteristics who were treated with surgery [2]. Increases in median survival has been explained by improving the efficacy of modern treatment of brain metastases. Patients with a single brain metastasis who receive surgical resection and post-operative WBRT experience a 25-week increase in medial overall survival, a 32% decrease in intracranial recurrence, and a 30-week increase in functional independence compared to patients treated with WBRT alone [16]. The results of our study show significant improvement in survival in patients treated with surgery combined with radiation in comparison to radiation alone on the Kaplan–Meier log-rank test and a trend toward this relationship for multivariate analysis.

However, our results showed that median survival post diagnosis of brain metastases is quite short (3.6 months). In keeping with our findings, for the largest study on this topic involving 853 patients with primary gynaecologic malignancies and brain metastases, median overall survival post diagnosis of brain metastases was 6.24, 4.99, and 4.34 months for patients with ovarian, cervical, and uterine cancer, respectively [11]. Further aligning with our findings, median overall survival noted by Ogawa et al. was 4.1 months in this patient population [1].

The short median survival observed (3.6 months) is likely related to the aggressive and advanced nature of the disease amongst our patient population, as evidenced by several clinicopathologic factors, including advanced staged disease (FIGO III/IV) (n = 75, 73%), high-grade tumours (grade 3) (n = 63, 61%), disseminated disease with ≥3 metastatic brain lesions (n = 55, 53%), and extracranial metastases (n = 80, 78%) at diagnosis of brain metastases. Moreover, there were a limited number of patients meeting criteria for surgical intervention, which is shown to improve survival [14,16].

However, it should be noted that while median survival was short, the survival range post diagnosis of brain metastases was quite broad extending from 0.4 to 183.8 months. Hence, there does remain a population of patients with the potential for prolonged survival after treatment, with the goal of univariate analysis (Table 2 and Table 3, Figure 2 and Figure 3) and multivariate analysis being to identify factors contributing to the survival differences seen in patients.

Cox regression showed that complete response to prior treatment was associated with a more favourable prognosis following diagnosis of brain metastases. Patients with a prior response to treatment may be more likely to respond to treatment for both brain metastases and extracranial disease, extending survival. As discussed above, treatment with a combination of surgery and radiation was additionally identified as being associated with prolonged survival. Alternatively, univariate and multivariate analysis showed that the presence of extracranial disease at diagnosis of brain metastases was associated with shorter survival for these patients with disseminated disease. Moreover, ≥3 brain metastases were noted to be a poor prognostic factor. This may be because patients with multiple brain metastases have more advanced disease in the brain, but also, they are less likely to be candidates for more effective treatments such as surgical intervention.

Overall, our study provides critical clinical information regarding prognosis and expected efficacy of treatment for patients and physicians managing these patients. Knowing that ≥3 brain metastases and the presence of extracranial disease tend to be associated with poorer prognosis, consideration may be made for less aggressive treatment of brain metastases in these patient groups, as despite best efforts at treatment, survival is shown to remain short. However, alternatively, we have shown that some patients lived up to 184 months following a diagnosis of brain metastases secondary to gynaecologic malignancy.

Hence, while brain metastases secondary to gynaecologic malignancy have classically been considered a poor prognostic factor overall, biasing physicians to a more palliative and/or less aggressive approach to treatment, some patients may have a prolonged survival, warranting a more aggressive treatment approach. Based on our results, patients for whom consideration should be made for a more aggressive treatment approach and who have the potential for prolonged survival with treatment would include those patients without a primary ovarian malignancy or disease outside of the brain, who have a limited number of metastatic brain lesions (i.e., 1–2), and who responded to prior treatment of their cancer. Our results additionally highlight the importance of ongoing use of modern treatment modalities such as surgery and SRS, where possible, which were associated with prolonged survival, while also known for reducing radiation toxicity.

Finally, we made an effort to investigate the incidence of brain metastases secondary to gynaecologic malignancy. While we did not have regional data regarding the number of patients with brain metastases or the total number of patients with gynaecologic malignancy available to us, our tertiary care centre services a large catchment area, the only centre in the region providing brain radiotherapy, and hence, we were able to use data from our centre as a surrogate for incidence calculations in the region. We observed a 37% rise in the incidence over 15 years. Rising incidence of brain metastases in this patient population has been previously documented [5,7]. In the study time frame, there have been significant changes in the treatment of advanced gynaecologic malignancies with the publication of landmark practice-changing trials, which have proven to result in significant improvements in progression-free and overall survival in patients with advanced high-risk gynaecologic malignancies [17,18,19,20,21]. Considering that brain metastases are shown to be a late phenomenon in gynaecologic malignancies, improved control of extracranial disease and prolonged survival in patients with advanced gynaecologic malignancy may explain the rise in the incidence of brain metastases overtime. Additional contributing factors may include increased use of head imaging to assess for brain metastases and increased detection of brain metastases with modern imaging. Regional or national registry data are required to confirm our incidence findings.

With the suggestion of a rising incidence of brain metastases secondary to gynaecologic malignancy, and considering that most patients in our study presented with neurological symptoms, clinicians should include brain metastases in the differential diagnosis of patients with gynaecologic malignancies with new neurologic symptoms. Early diagnosis of brain metastases will prompt sooner referral to radiation oncology to facilitate earlier treatment of brain metastases, which may improve patient outcomes.

The strengths of this study were the large cohort size, the collection of numerous clinicopathological factors for assessment, and real-world survival data in this unique population over a two-decade period of time. The retrospective design and data incompleteness remains a weakness, and the single-institution nature of this study limits external validity; however, it increases consistency of treatment amongst subjects and increases the accuracy of data collection. The long period of time (two decades) has the potential to introduce bias with changing treatment options and diagnostic modalities; however, considering the rarity of brain metastases secondary to gynaecologic malignancy, it was required to obtain larger study numbers. One further weakness is that the incidence calculations are not based on national or regional data; however, we are accepting of the limitations in the accuracy of true incidence in favour of reporting a measure of the change in frequency of brain metastases secondary to gynaecologic malignancy overtime.

## 5. Conclusions

Our study is a large, single-centre study showing that median survival remains poor post diagnosis of brain metastases (3.6 months) in a patient population with particularly advanced gynaecologic cancer. However, median survival post diagnosis of brain metastases and median overall survival are significantly longer for patients who received surgery combined with radiation compared to radiation alone, and SRS compared to WBRT, providing an indication for these treatment modalities in this patient population. Further, we were able to identify clinicopathologic factors associated with poorer or more favourable prognosis. These findings are clinically relevant, providing important information to physicians and patients regarding prognosis post diagnosis of brain metastasis, which helps to set expectations regarding survival and may also impact treatment decision making. Large multi-centre future studies are necessary to investigate this topic further.

## Figures and Tables

**Table 1:** Patient clinical characteristics.

					n (%)					
Clinical Measures	Overall		Uterus		Ovary		Cervix		Vagina/Vulva	
Stage at initial cancer diagnosis										
FIGO I/II	25	(24%)	14	(14%)	5	(5%)	4	(4%)	2	(2%)
FIGO III/IV	75	(73%)	20	(19%)	41	(40%)	12	(12%)	2	(2%)
N/A	3	(3%)	0	(0%)	0	(0%)	0	(0%)	3	(3%)
Tumour grade at initial cancer diagnosis										
1	4	(4%)	4	(4%)	0	(0%)	0	(0%)	0	(0%)
2	14	(14%)	8	(8%)	4	(4%)	2	(2%)	0	(0%)
3	63	(61%)	19	(18%)	40	(39%)	4	(4%)	0	(0%)
N/A	22	(21%)	3	(3%)	2	(2%)	10	(10%)	7	(7%)
Brain metastases at initial cancer diagnosis										
No	98	(95%)	31	(30%)	44	(43%)	16	(16%)	7	(7%)
Yes	5	(5%)	3	(3%)	2	(2%)	0	(0%)	0	(0%)
Best response to prior treatments										
Complete response	62	(60%)	21	(20%)	32	(31%)	6	(6%)	3	(3%)
Partial response	32	(31%)	8	(8%)	11	(11%)	9	(9%)	4	(4%)
Progressive Disease	3	(3%)	2	(2%)	1	(1%)	0	(0%)	0	(0%)
N/A (no prior treatment)	6	(6%)	3	(3%)	2	(2%)	1	(1%)	0	(0%)
Initial imaging modality for brain metastases diagnosis										
CT	84	(82%)	28	(27%)	42	(41%)	10	(10%)	4	(4%)
MRI	19	(18%)	6	(6%)	4	(4%)	6	(6%)	3	(3%)
Symptoms at time of brain metastases diagnosis										
No	6	(6%)	2	(2%)	2	(2%)	1	(1%)	1	(1%)
Yes	97	(94%)	32	(31%)	44	(43%)	15	(15%)	6	(6%)
No. brain metastases at diagnosis of brain metastases										
1	34	(33%)	13	(13%)	14	(14%)	6	(6%)	1	(1%)
2	14	(14%)	2	(2%)	9	(9%)	2	(2%)	1	(1%)
≥3	55	(53%)	19	(18%)	23	(22%)	8	(8%)	5	(5%)
Extracranial metastases at brain metastases diagnosis										
No	23	(22%)	6	(6%)	14	(14%)	3	(3%)	0	(0%)
Yes	80	(78%)	28	(27%)	32	(31%)	13	(13%)	7	(7%)
Site of extracranial metastases										
Abdomen/pelvis	63	(61%)	20	(19%)	29	(28%)	9	(9%)	5	(5%)
Chest	50	(49%)	22	(21%)	14	(14%)	7	(7%)	7	(7%)
Bone	18	(17%)	9	(9%)	4	(4%)	3	(3%)	2	(2%)
N/A—no extracranial metastases	23	(22%)	6	(6%)	14	(14%)	3	(3%)	0	(0%)

**Table 2:** Median survival post diagnosis of brain metastases and overall survival based on clinicopathological factors with univariate analyses by the Kaplan–Meier method log-rank test.

	Months (95% CI)					
	Median Survival Post Diagnosis of Brain Metastases		*p* Value	Median Overall Survival		*p* Value
Site of primary gynaecologic malignancy			*p* = 0.92			*p* = 0.214
Uterine	1.7	(1.2, 2.1)		24.9	(18.9, 30.9)	
Ovary	5.1	(0.9, 9.2)		45.4	(34.6, 56.2)	
Cervix	5.3	(2.5, 8.1)		16.5	(0, 35.0)	
Vagina/Vulva	5.1	(2.2, 4.9)		24.2	(0, 53.6)	
Stage at initial cancer diagnosis			*p* = 0.390			*p* = 0.252
FIGO I/II	3.2	(0, 7.6)		45.4	(40.5, 50.4)	
FIGO III/IV	3.6	(2.2, 4.9)		27.9	(16.0, 39.8)	
Tumour grade at initial cancer diagnosis			*p* = 0.147			*p* = 0.292
1/2	1.6	(0.7, 2.5)		31.0	(21.5, 40.4)	
3	4.4	(2.7, 6.1)		37.6	(29.2, 45.94)	
N/A	5.1	(2.8, 7.3)		23.3	(4.1, 42.5)	
Brain metastases at initial cancer diagnosis			*p* = 0.002			*p* < 0.001
No	4.0	(2.5, 5.6)		37.3	(28.2, 46.3)	
Yes	1.6	(0.1, 3.1)		2.0	(0.39, 3.7)	
Best response to prior treatments			*p* < 0.001			*p* < 0.001
Complete response	5.3	(1.8, 8.8)		44.1	(37.1, 51.1)	
Partial response	3.3	(2.2, 4.5)		18.7	(7.1, 30.3)	
Progressive Disease	1.0	(0.9, 1.1)		8.3	(4.6, 12.1)	
N/A (no prior treatment)	1.0	(0.1, 1.8)		2.0	(1.0, 3.0)	
No. brain metastases at brain metastases diagnosis			*p* < 0.001			*p* < 0.001
1/2	7.2	(2.8, 11.7)		47.5	(40.6, 54.3)	
≥3	2.7	(1.7, 3.7)		24.4	17.2, 31.6)	
Extracranial metastases at brain metastases diagnosis			*p* < 0.001			*p* = 0.002
No	16.7	(7.0, 26.4)		47.1	(38.3, 56.0)	
Yes	2.7	2.1, 3.4)		24.5	(16.7, 32.3)	
Date of treatment of brain metastases			*p* = 0.335			*p* = 0.100
January 2004–December 2013	3.2	(1.4, 5.0)		30.4	(22.1, 38.7)	
January 2014–September 2023	4.0	(2.0, 6.0)		37.7	(20.9, 54.5)	

**Table 3:** Median survival post diagnosis of brain metastases and overall survival based on treatment provided, with univariate analyses by the Kaplan–Meier method log-rank test.

	Months (95% CI)					
	Median Survival Post Diagnosis of Brain Metastases		*p* Value	Median Overall Survival		*p* Value
Treatment of Brain Metastasis			*p* < 0.001			*p* < 0.001
Radiation alone	2.6	(1.6, 3.5)		24.5	(15.6, 33.4)	
Radiation + surgery	13.3	(0.5, 26.1)		67.5	(42.0, 93.0)	
Type of Radiation Provided			*p* < 0.001			*p* < 0.001
WBRT	2	(1.2, 2.9)		24.9	(15.2, 34.6)	
SRS	8.9	(6.3, 11.4)		67.5	(37.7, 97.4)	
WBRT + SRS	7	(5.4, 8.7)		37.6	(0, 82.0)	

**Table 4:** Estimated incidence of brain metastases secondary to gynaecologic malignancy.

Year	Referral to Radiation Oncology for Diagnosis of Brain Metastases Secondary to Gynaecologic Malignancy (n)	Referral for Diagnosis of Gynaecologic Malignancy (n)	Estimated Incidence (%)
2004–2008	17	N/A	N/A
2009–2013	21	1672	1.3
2014–2018	32	1986	1.6
2019–2023	33	1916	1.7

## Data Availability

Data are contained within the article.
